# In Silico Approach for Antibacterial Discovery: PTML Modeling of Virtual Multi-Strain Inhibitors Against *Staphylococcus aureus*

**DOI:** 10.3390/ph18020196

**Published:** 2025-01-31

**Authors:** Valeria V. Kleandrova, M. Natália D. S. Cordeiro, Alejandro Speck-Planche

**Affiliations:** LAQV@REQUIMTE/Department of Chemistry and Biochemistry, Faculty of Sciences, University of Porto, 4169-007 Porto, Portugal; valeria.kleandrova@gmail.com (V.V.K.); ncordeir@fc.up.pt (M.N.D.S.C.)

**Keywords:** PTML, topological indices, multilayer perceptron, subgraph, fragment, fragment-based topological design, antibacterial

## Abstract

**Background/Objectives**: Infectious diseases caused by *Staphylococcus aureus* (*S. aureus*) have become alarming health issues worldwide due to the ever-increasing emergence of multidrug resistance. In silico approaches can accelerate the identification and/or design of versatile antibacterial chemicals with the ability to target multiple *S. aureus* strains with varying degrees of drug resistance. Here, we develop a perturbation theory machine learning model based on a multilayer perceptron neural network (PTML-MLP) for the prediction and design of versatile virtual inhibitors against *S. aureus* strains. **Methods**: To develop the PTML-MLP model, chemical and biological data associated with antibacterial activity against *S. aureus* strains were retrieved from the ChEMBL database. We applied the Box–Jenkins approach to convert the topological indices into multi-label graph-theoretical indices; the latter were used as inputs for the creation of the PTML-MLP model. **Results**: The PTML-MLP model exhibited accuracy higher than 80% in both training and test sets. The physicochemical and structural interpretation of the PTML-MLP model was performed through the fragment-based topological design (FBTD) approach. Such interpretations permitted the analysis of different molecular fragments with favorable contributions to the multi-strain antibacterial activity and the design of four new drug-like molecules using different fragments as building blocks. The designed molecules were predicted/confirmed by our PTML model as multi-strain inhibitors of diverse *S. aureus* strains, thus representing promising chemotypes to be considered for future synthesis and biological testing of versatile anti-*S. aureus* agents. **Conclusions**: This work envisages promising applications of PTML modeling for early antibacterial drug discovery and related antimicrobial research areas.

## 1. Introduction

Bacterial infections are widely recognized as life-threatening medical conditions, being associated with high morbidity and mortality rates. A recent epidemiology-based analysis estimated that, in 2019, bacterial infections were related to 13.7 million deaths, with more than 50% of them involving 33 bacteria [1]. Currently, among these pathogenic microorganisms, *Staphylococcus aureus* (*S. aureus*) represents a great concern to public health, being the only pathogenic bacterium (in addition to *Mycobacterium tuberculosis*), whose mortality exceeds 1 million deaths annually [1]. Common and widespread infections caused by *S. aureus* include (but are not limited to) skin infections, pneumonia and other respiratory tract infections, cardiovascular infections, and nosocomial bacteremia [2]. Over time, infections caused by *S. aureus* have become increasingly difficult to treat, mainly because of the emergence of the phenomenon known as multidrug resistance (MDR), which can be either developed or acquired [3]. Furthermore, despite being poorly understood, zoonosis can contribute to MDR because different *S. aureus* strains may cross the interspecies barriers, thus exhibiting new patterns and mechanisms of intrinsic MDR [4]. All this indicates that, finding efficacious antibacterial agents against *S. aureus* represents an imperative need.

Nowadays, although experimental methods in drug discovery remain the gold standard for validation/identification of new therapeutic solutions against any disease (including infections caused by *S. aureus*), these are complex, expensive, and time-consuming, and should therefore be guided and rationalized by the use of computational approaches [5,6]. In the context of antibacterial drug discovery against *S. aureus*, a series of computational approaches involving density functional theory [7,8], pharmacophore modeling [9], structure-based drug discovery (molecular docking and molecular dynamic simulations) [7,8,9,10,11,12,13,14], machine learning algorithms focused on quantitative structure–activity relationships (QSAR) for modeling and virtual screening [15,16,17]. Yet, all these methods present at least one of the following major limitations: (a) the use of small chemical datasets of structurally related chemicals (preventing a wider exploration of the chemical space), (b) the prediction of biochemical activity (which doesn’t necessarily translate into an effective phenotypic effect against *S. aureus*), (c) the no specification of the *S. aureus* strains against which the antibacterial activity is predicted/modeled (being this factor detrimental when attempting to find versatile inhibitors of multiple drug-resistant strains), and (d) the lack of interpretation physicochemical and structural (insufficient information halting the computer-aided de novo design of new molecules).

The past decade has seen the emergence and consolidation of the in silico approach known as perturbation theory machine learning (PTML) [18,19]; this approach has overcome all the aforementioned limitations. In this sense, PTML models are advanced two-dimensional QSAR (2D-QSAR) models capable of integrating chemical and biological information at different levels of complexity and diversity, allowing the simultaneous prediction of multiple endpoints against different biological targets (e.g., proteins, microbes, cell lines, etc.) and across a wide array of assay protocols [18,19]. Applications of PTML modeling have been reported in the context of nanotechnology for drug delivery [20,21,22], neurosciences [23,24,25], biosequence-based bioactive molecules (e.g., peptides and epitopes) [26,27,28,29], immunotoxicity [30,31], anticancer drug discovery [32,33,34,35,36,37], and antimicrobial research [38,39,40,41,42,43,44,45]. However, there has been no report focusing on the computer-aided rational design of potentially novel and versatile inhibitors of *S. aureus* strains. In this work, we establish the theoretical foundations for the application of PTML modeling to early antibacterial drug discovery against *S. aureus*. Particularly, we report for the first time, a PTML model based on a multilayer perceptron network (PTML-MLP) for the prediction of multi-strain antibacterial activity of molecules against *S. aureus*. Through the application of the fragment-based topological design (FBTD) approach we demonstrate that it is possible to physicochemically and structurally interpret the PTML-MLP model [19,46], thus leading to the design of four new drug-like chemicals virtually exhibiting multi-strain antibacterial activity against *S. aureus*.

## 2. Results and Discussion

### 2.1. The PTML-MLP Model

The most appropriate PTML-MLP model found by us had the notation MLP 21-72-2, which means that 21 *D*[*GTI*]*bs* descriptors were used as input nodes *I_n_*. The PTML-MLP model also contained *H_n_* = 72 (hidden neurons) and *O_n_* = 2 (output nodes). The PTML-MLP model used logistic and hyperbolic tangent as the activation functions in the hidden and output layers, respectively. Considering that *T_c_* = 8738 training cases (see Supplementary Information S1), we applied the following mathematical formalism:

(1) $$\rho =\frac{T_{c}}{\left[(I_{n}+1)H_{n}+{(H}_{h}+1)O_{n}\right]}$$

Notice that Equation (1) considers the adequacy of the topology of a multilayer perceptron network (in this case, our PTML-MLP model). To prevent the neural network from overfitting the date, the parameter *ρ* should be expected to reach a value higher than 3 [47,48]. In this work, we obtained the value of *ρ* = 5.051; because *ρ* > 3, we can conclude that our PTML-MLP model is not overfitting the data [47,48]. The concepts associated with the 21 *D*[*GTI*]*bs* descriptors present in the PTML-MLP model are depicted in Table 1; details on the different physicochemical properties (e.g., hydrophobic, polar, and steric factors) and multiple structural moieties that can influence the appearance/enhancement of the multi-strain antibacterial activity against *S. aureus* are given in the next subsection.

The PTML-MLP model reported in this work exhibits good performance; the *Acc* values are 88.02% and 82.51% in the training and test sets, respectively. Furthermore, Table 2, shows the number of molecules/cases annotated as active and inactive (*N*_Active_ and *N*_Inactive_, respectively), as well as other statistical indices.

In Table 2, it can be seen that there is a high number of correctly classified active (*CC*_Active_) and inactive (*CC*_Inactive_) cases. This translates into *Sn* > 84% and *Sp* > 90% in the training set, indicating the good internal statistical quality of the PTML-MLP model. At the same time, the PTML-MLP model also achieved *Sn* around 80% and *Sp* higher than 84% in the test set, thus demonstrating adequate predictive power. The appropriate performance of the PTML-MLP model is also confirmed through the analysis of the *nMCC* values; these are closer to 1, which indicates that there is a strong agreement (correlation) between the observed [*ABi*(*bs*)] and the predicted [*Pred*-*ABi*(*bs*)] values of antibacterial activity. Detailed information concerning the classification results for each of the 11,643 cases in our dataset can be found in Supplementary Information S2.

In any case, a major advantage of our PTML-MLP model is its ability to simultaneously classify/predict molecules against different *S. aureus* strains (*bs*). In this sense, when analyzing the local sensitivity *Sn*(*bs*) and specificity *Sp*(*bs*) values (Supplementary Information S2), one can see, that, in the training set, *Sn*(*bs*) is in the interval 75–95% while *Sp*(*bs*) in the range 78–98%; the only exception is *S. aureus* (N315), for which *Sn*(*bs*) = 60%. In the test set, *Sn*(*bs*) and *Sp*(*bs*) exhibited similar tendencies as in the training set, being both in the interval 75–95%. The exceptions were only 4 out of 13 the *S. aureus* strains (*bs*) for which *Sn*(*bs*) or *Sp*(*bs*) [never both] was below 70%: ATCC 25923 [*Sn*(*bs*) = 67.18%], ATCC 33591 [*Sp*(*bs*) = 62.00%], ATCC 33592 [*Sp*(*bs*) = 68.00%], and ATCC 25923 [*Sn*(*bs*) = 46.67%]. Altogether, the *Sn*(*bs*) and *Sp*(*bs*) values demonstrate the capabilities of the PTML-MLP model to predict antibacterial activity across multiple *S. aureus* strains.

Regarding the applicability domain (AD) of the PTML-MLP model, we applied a modification of the bounding box approach. In doing so, we calculated the so-called local scores of the applicability domain (LSAD) according to recent reports [37,45]. Each LSAD was calculated for each of the *D*[*GTI*]*bs* descriptors present in the PTML-MLP model. Thus, for each *D*[*GTI*]*bs* descriptor, the minimum and maximum values (considering only the correctly classified molecule/cases in the training set) were computed. If for a given *D*[*GTI*]*bs* descriptor, a molecule/case had a *D*[*GTI*]*bs* descriptor value within the minimum and maximum values for that particular *D*[*GTI*]*bs* descriptor, the LSAD was equal to one; otherwise, the LSAD took the value of zero. This procedure was applied to each *D*[*GTI*]*bs* descriptor and each of the 11,643 molecules/cases. Because there were 21 *D*[*GTI*]*bs* descriptors present in the PTML-MLP model, 21 LSAD values for each molecule were calculated. A molecule/case was considered to be within the AD of the PTML-MLP model if all its LSAD values were equal to 1 (i.e., the sum of the LSAD values was equal to 21); otherwise, the molecule/case was considered to be out of the AD. In our dataset, 11,630 out of 11,643 molecules/cases fell within the AD of the PTML-MLP model (Supplementary Information S2).

**Figure 1:**
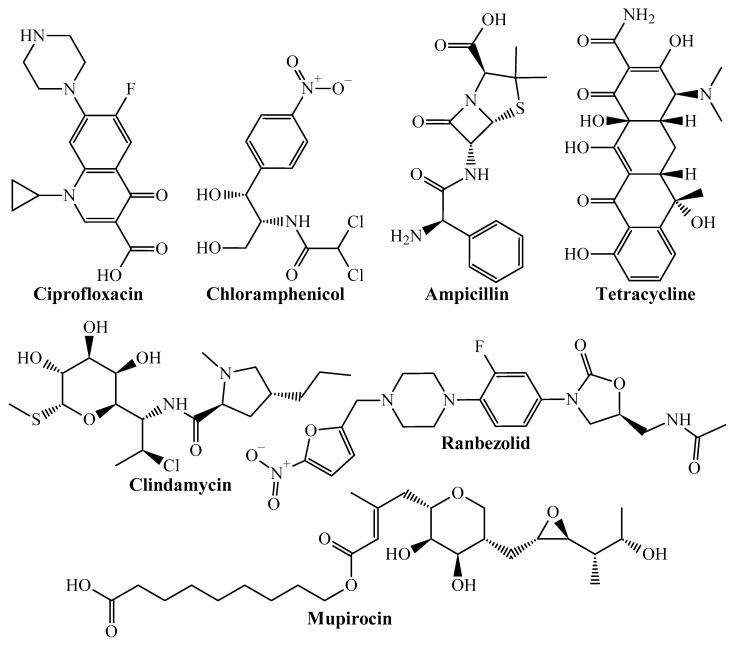
Non-exhaustive list of experimental and FDA-approved antibacterial drugs correctly predicted by the PTML-MLP model as multi-strain inhibitors against *S. aureus*.

The chemical structures depicted in Figure 1 are known antibacterial agents and are reported with determined experimental minimum inhibitory concentration (MIC) values in our dataset used to build the PTML-MLP model.

**Figure 2:**
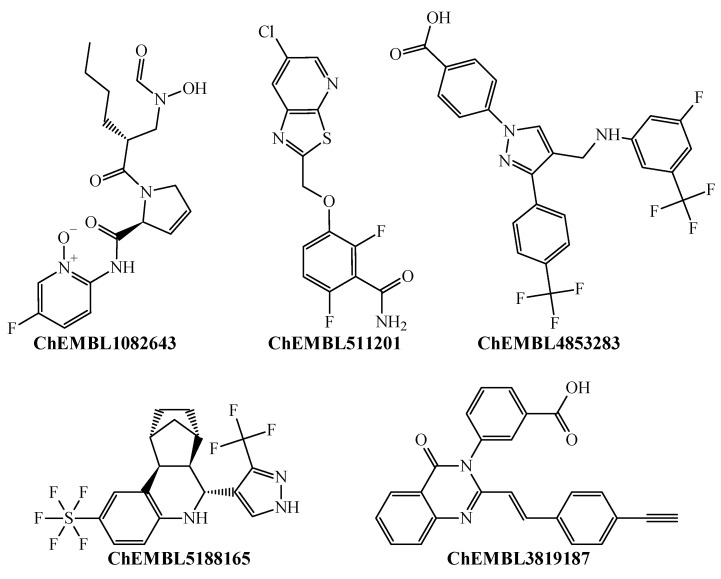
Top molecules representing new chemotypes that were experimentally tested and correctly predicted as multi-strain inhibitors.

Our PTML-MLP model could accurately predict the multi-strain antibacterial activity of these antibacterial drugs, which are either approved by the Food and Drug Administration (FDA) or at different experimental stages. This demonstrates, that, our PTML-MLP model, in addition to having the capability of predicting antibacterial activity chemicals across 13 different *S. aureus* strains (*bs*) exhibiting different degrees of antibiotic resistance, can also detect privileged molecular patterns as those belonging to the antibacterial drugs illustrated in Figure 1. On the other hand, our PTML-MLP model could also correctly predict other molecules (Figure 2).

These chemical structures are considerably different from the experimental/FDA-approved antibacterial drugs. These molecules are also experimentally reported in our dataset as multi-strain inhibitors. By correctly predicting them, our PTML-MLP model has confirmed its capacity to identify new chemotypes that can target multiple *S. aureus* strains, thus constituting promising alternatives to be considered in future antibacterial discovery and development to tackle multidrug resistance.

We would like to point out that although our PTML-MLP model has very good capabilities to simultaneously predict antibacterial activity against multiple *S. aureus* strains, it also presents two main limitations. One of them is, that, the descriptors (in our case, the *D*[*GTI*]*bs* descriptors) are not able to fully characterize the chemical diversity and complexity of the dataset used to build it. This is a common problem in descriptors-based machine learning models, which indicates that even combinations of different molecular descriptors can only account for the reduced fraction of information present in datasets [49,50,51]. This limitation helps explain those chemicals incorrectly predicted/classified by the PTML-MLP model which translated into very good but not optimum values of the global (*Sn* and *Sp*) and local [*Sn*(*bs*) and *Sp*(*bs*)] statistical indices. This also means that our PTML-MLP model, like any machine learning model, will be able to perform accurate prediction to a certain extent because the chemical space contained in the dataset is considerably smaller than the vast one that is available for virtual screening. Such inaccuracies in predictive power could be detrimental to the future experimental validation of new molecules predicted from virtual screening scenarios. This limitation can be at least partially mitigated by resorting to the fragment-based topological design (FBTD) [37,45,52,53], which, as will be seen in the upcoming subsections, instead of selecting/identifying compounds via virtual screening, offers a direct physicochemical and structural interpretation of the PTML-MLP model, thus subsequently allowing the design of new molecules potentially increased synthetically accessibility and considerably higher probabilistic scores to be active (in the case of the present study, higher probability to be multi-strain inhibitors). The second limitation of our PTML-MLP model is related to the lack of characterization of stereochemistry. In this sense, different stereoisomers (e.g., enantiomers) can either lead to very different results in terms of biological activity or, in other cases, the difference is negligible from a therapeutic point of view. While stereochemistry is a critical factor in drug discovery, the scope of this study focuses on predicting the multi-strain antibacterial activity of molecules based on descriptors that primarily capture 2D molecular patterns. As will be described in the upcoming Section 2.2, the *D*[*GTI*]*bs* descriptors used to build the PTML-MLP model contain considerable physicochemical and structural information (described by the FBTD approach), thus rationalizing the detection/identification/computer-aided design of 2D molecular patterns and subsequently reducing the number of chemical structures containing stereoisomers.

### 2.2. Designing Multi-Strain Inhibitors Through the FBTD Approach

#### 2.2.1. Interpreting the Multi-Label Graph-Theoretical Indices

**Figure 3:**
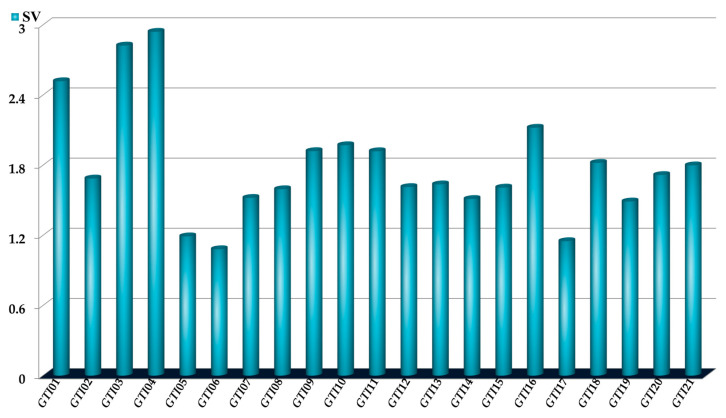
Relative influence of the *D*[*GTI*]*bs* descriptors in the PTML-MLP model assessed by the *SVs*. The codes used in the illustration are the same as the ones represented in Table 1.

Any PTML model can in principle be physicochemically and structurally interpreted. When the inputs of a PTML model are multi-label graph-theoretical indices (as the *D*[*GTI*]*bs* descriptors reported in this work in Table 1), one can apply the FBTD approach to gather information from the multi-label graph-theoretical indices in the sense of analyzing the physicochemical properties and structural features are can favorably influence the biological activity under study (in our case) [37,45,52,53]. When applying the FBTD approach in this work, two steps have been considered, namely (a) the physicochemical and structural interpretations of each *D*[*GTI*]*bs* descriptor in the PTML-MLP model, allowing the identification and analysis of different molecular fragments and (b) the use of the aforementioned interpretation to design new molecules by fusing/connecting different molecular fragments with positive influence on the multi-strain antibacterial activity. For the first steps, we have calculated the sensitivity values (*SVs*), which are illustrated in Figure 3.

**Figure 4:**
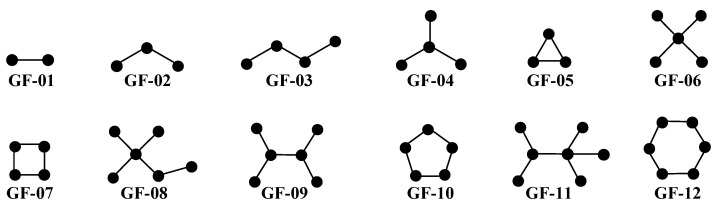
Different subgraphs, which, as molecular fragments, are expected to have a favorable influence on the increase in the multi-strain antibacterial activity.

The *SVs* assess the degree of importance/discriminatory power and the information content of each *D*[*GTI*]*bs* descriptor in the PTML-MLP model. In this sense, we would like to highlight that the *SVs* rank the importance of the model’s input variables [54]. In the context of the present work, the *SVs* quantitatively assess the relative importance of the *D*[*GTI*]*bs* descriptors. The highest *SVs* are associated with those *D*[*GTI*]*bs* descriptors with the greatest discriminatory power, which means that the physicochemical properties and structural features characterized by them should be present in both the dataset used to build the PTML-MLP model and any molecule to be designed. For the case of the information content, it is important to take into account, that, the *D*[*GTI*]*bs* descriptors maintain the same physicochemical and structural meaning as the original topological indices [*SM*(*A*)*m*, *X*(*GF*)*k*, *Xv*(*GF*)*k*, *e*(*GF*)*k*, and *K*(*Alpha*)*t*] from which the *D*[*GTI*]*bs* descriptors were calculated (see Equations (1) and (2) in Section 3). When interpreting the *D*[*GTI*]*bs* descriptors, we will describe how their values will favorably vary to increase the multi-strain antibacterial activity (Table 3) [37,45,52]. We will also describe the subgraphs (*GF*s) involved in such variations (Figure 4); since the *GFs* are generic fragments, we will also mention different molecular fragments (e.g., polar functional groups aliphatic chains, aliphatic and aromatic rings, presence of halogens, etc.) representing the *GFs*.

It is essential to emphasize that when interpreting the *D*[*GTI*]*bs* descriptors, one should not expect the values of the *D*[*GTI*]*bs* descriptors to be increased or decreased infinitely. This comes from the fact that the values of the *D*[*GTI*]*bs* descriptors have their boundaries (i.e., minimum and maximum values estimated by the AD of the PTML-MLP model discussed above). At the same time, the physicochemical and structural information in a defined *D*[*GTI*]*bs* descriptor is usually constrained by one or more *D*[*GTI*]*bs* descriptors; consequently, the number of molecular fragments (derived from those subgraphs *GFs*) is neither expected to vary (increase or decrease) infinitely.

For instance, 11 out of the 21 *D*[*GTI*]*bs* descriptors are derived from the topological indices known as the bond spectral moments *SM*(*A*)*m*. In this sense, it is important to say that *SM*(*A*)*m* (and consequently, the 11 *D*[*GTI*]*bs* descriptors derived from them) describe the concentration of any physicochemical property in fragments of different sizes in a molecule and can be expressed as a linear combination of the number of times in which those fragments appear in a molecule [55,56,57,58,59,60]. Therefore, it is possible to have information on how different regions within a molecule can positively or negatively contribute to the multi-strain antibacterial activity.

In this work, despite its simplicity, GF-01 (considering each bond of a molecule) is associated with global physicochemical properties such as the increment of the polar surface area (characterized by *DGTI*01), the augmentation of the polarizability (described by *DGTI*03), and the diminution of the hydrophobicity (*DGTI*13 and *DGTI*18). The descriptors *DGTI*01, *DGTI*03, *DGTI*13, and *DGTI*18 rank third, second, twelfth, and eighth, respectively. Altogether, the best way to favorably and simultaneously vary the values of these four *D*[*GTI*]*bs* descriptors is to increase the presence of pyridinic nitrogen atoms, and thus, the presence of heteroaromatic rings (imidazole, oxazole, pyridine, pyrazine, etc.). In addition to GF-01, GF-02 is also a simple, yet important subgraph mainly associated with steric factors, which are characterized by the descriptors *DGTI*11, *DGTI*015, and *DGTI*016 (respectively, ranked as the seventh, fourteenth, and fourth most important descriptors in the PTML-MLP model). On one side, *DGTI*11 involves the increment of the bond distance while *DGTI*16 describes the increase in the van der Waals radius of the atoms. The joint interpretation of *DGTI*11 and *DGTI*16 suggests that specific single bonds may also be important, particularly those involving halogens other than fluorine. On the other hand, *DGTI*015 supports the decrease in the polarizability, thus favoring the presence of low-polarizability functional groups (e.g., amides and moieties containing fluorine) as well as heteroaromatic rings containing two heteroatoms separated by no more than two bonds (without counting multiplicity) such as azoles, pyrimidine, and pyridazine. Other key subgraphs are GF-04 and GF-06; they are contained in the information characterized by the augmentation of the polar surface area (*DGTI*02, ranked eleventh), the increase of the polarity of the bonds (*DGTI*12, ranked thirteenth), the increase in hydrophobicity (*DGTI*14, ranked seventeenth), and the diminution of the atomic weight (*DGTI*17, ranked twentieth); in addition, *DGTI*12 and *DGTI*14 are associated with other important subgraphs such as GF-08 and GF-09 (with *DGTI*12 also considering GF-09 desirable). Favorably varying all these descriptors at the same time can mainly be achieved by the presence of a trifluoromethyl group and/or incrementing the number of substitutions in rings by using four-atoms polar groups associated with GF-04 (e.g., amide).

The PTML-MLP model also contains six *D*[*GTI*]*bs* descriptors derived from the bond connectivity indices *e*(*GF*)*k*, which means that they are quantitative measures of the molecular volume in different regions/subgraphs/fragments within a molecule [61,62,63,64,65]. These *D*[*GTI*]*bs* descriptors are *DGTI*05, *DGTI*06, *DGTI*07, *DGTI*08, *DGTI*09, and *DGTI*21; these rank nineteenth, twenty-first, sixteenth, fifteenth, sixth, and ninth among the most discriminant in the PTML-MLP model, respectively. Here, these *D*[*GTI*]*bs* descriptors indicate the increase in the number of subgraphs of the type GF-05 (three-membered rings accounted for by *DGTI*05), GF-07 (four-membered rings considered by *DGTI*06), GF-10 (five-membered rings described by *DGTI*07), and GF-12 (six-membered rings characterized by *DGTI*09). On the other hand, *DGTI*08 involves the increment molecular volume as a consequence of the increase in the number of GF-11 subgraphs. Such an increase in the value of *DGTI*08 favors the presence of structural moieties where functional groups such as trifluoromethyl, sulfone, sulfonamide, and tert-butyl are attached to any ring; it is important to highlight that a ring should not contain any substitution in the positions adjacent to the one in which any of the aforementioned groups is placed. In the case of *DGTI*09, this characterized the diminution of the global volume of the molecule (GF-01 subgraph), thus being directly associated with the increase in the number of ramifications in a molecule (including polysubstituted and condensed rings).

There are also three *D*[*GTI*]*bs* descriptors derived from the atom-based connectivity indices *X*(*GF*)*k* and *Xv*(*GF*)*k*, which means that these *D*[*GTI*]*bs* descriptors measure the molecular accessibility [66,67,68,69,70,71,72], i.e., the ability different regions/fragments of a molecule to be available to interact with the surrounding media (e.g., solvent molecules). In this sense, we have the descriptor *DGTI*04, which indicates the increment of GF-04 subgraphs, thus increasing the number of ramifications through both polar (amide, sulfone, sulfoxide, sulfonamide, trifluoromethyl, etc.) or non-polar (isopropyl or tert-butyl); the present of substituted rings (at least in two positions) and condensed systems is also desirable. We would like to highlight that *DGTI*04 is the most important *D*[*GTI*]*bs* descriptor in the PTML-MLP model. On the other hand, *DGTI*19 (the eighteenth most important descriptor) suggests that the molecular accessibility in GF-02 subgraphs should be reduced; this can be achieved by diminishing as much as possible the number of atoms that do not belong to the second period of the periodic table. Therefore, the structure of a molecule should be focused more on functional groups and moieties containing carbon, oxygen, nitrogen, and fluorine. If an atom beyond the second period (e.g., chlorine or sulfur) is present, it should be preferably attached to an aromatic ring rather than present in aliphatic portions. For the case of the descriptor *DGTI*20, one should decrease the molecular accessibility of GF-12 subgraphs (six-membered rings), thus prioritizing the presence of aromatic rings (including those with heteroatoms) over their aliphatic counterparts.

Lastly, we have *DGTI*10, which has the tenth most significant influence on the PTML-MLP model. It is important to point out that *DGTI*10 is derived from a shape index, characterizing the increment of the linearity of the molecule by increasing the number of GF-03 subgraphs [73]. This descriptor restricts the increment of cyclic fragments (rings) and ramifications; if the latter are present, they are preferred in the periphery of the molecule. The presence of short non-cyclic aliphatic portions favorably increases the value of the descriptor *DGTI*10 (the fifth most important in the PTML-MLP model).

#### 2.2.2. Designing Multi-Strain Inhibitors Against *S. aureus*

**Figure 5:**
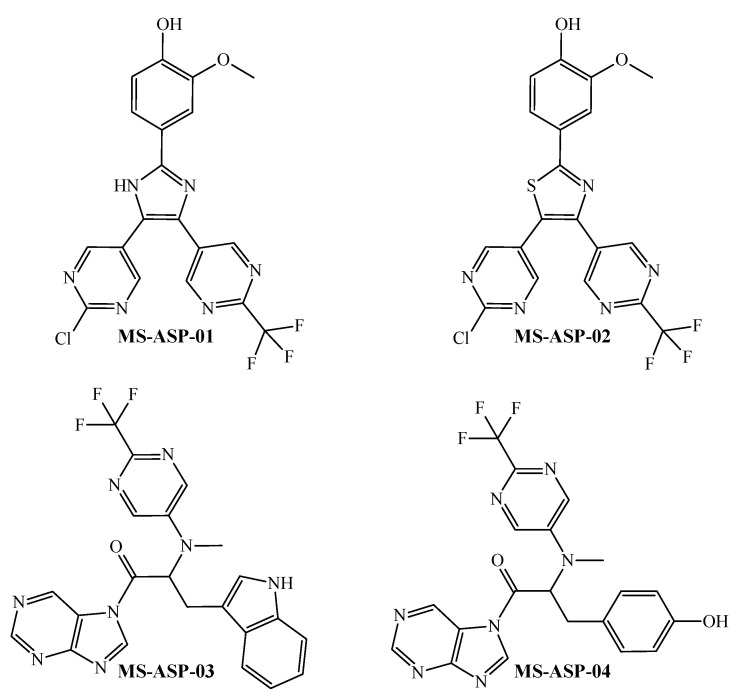
Chemical structures of the molecules designed to act as multi-strain antibacterial agents against *S. aureus*.

To exert multi-strain antibacterial activity against *S. aureus*, the joint interpretation of all the *D*[*GTI*]*bs* descriptors suggests that at least two or three six-membered heteroaromatic rings (containing each at least two pyridinic nitrogen atoms) should be present; this is valid for six-membered heteroaromatic rings either alone or as a part of condensed systems. The presence of an azole ring is also a desirable feature that a molecule should have. Both azoles and six-membered heteroaromatic rings should be substituted in at least two positions. Substituents to be present in any of these rings are trifluoromethyl, certain polar groups such as amides, and halogens (if different from fluorine, only one halogen is preferred). Short aliphatic portions can be beneficial, particularly when attached to polar groups or electronegative atoms such as nitrogen or oxygen. We designed four molecules, which are depicted in Figure 5.

We would like to emphasize that these molecules were designed by connecting and/or fusing different fragments whose presence is expected to enhance the multi-strain antibacterial activity; this is the second step regarding the application of the FBTD approach.

The fragments mentioned in the joint interpretation of the *D*[*GTI*]*bs* descriptors are the ones whose presence favorably varies the values of more than one *D*[*GTI*]*bs* descriptor. Such fragments are also associated with the most discriminant *D*[*GTI*]*bs* descriptors. Consequently, the designed molecules contained all the aforementioned characteristics. Among the four designed molecules MS-ASP-01 and MS-ASP-02 have great structural similarity; the same is valid for MS-ASP-03 and MS-ASP-04. The idea here was to analyze the effect of small variations in terms of physicochemical properties and structural features. We employed our PTML-MLP model to assess the multi-strain antibacterial activity of the designed molecules. A summary and details of the prediction results can be found in Table 4 and Supplementary Information S3, respectively.

Each value in Table 4 is the predicted probability [ProbAct] for a molecule to be considered active against a defined *S. aureus* strain. This means that if a ProbAct value for a molecule against an *S. aureus* strain was higher than 50%, then, the molecule was classified as active against that strain, thus potentially exhibiting MIC ≤ 7000 nM (the antibacterial activity cutoff used in this work to build the PTML-MLP model and described in detail in Section 3). The ProbAct values suggest that the four designed molecules are active against at least 10 of the 13 *S. aureus* strains; therefore, these designed molecules can be considered multi-strain inhibitors against *S. aureus*. Yet, there are some differences among these designed molecules. This also means that, at the theoretical level, the fragments used as building blocks, in addition to being correctly connected/fused according to the FBTD approach to yield designed molecules exhibiting high antibacterial versatility against the 13 *S. aureus* strains, may have an important influence on the appearance/enhancement of the multi-strain antibacterial activity. For instance, if we compare MS-ASP-01 and MS-ASP-02, we can see that the former is predicted to be active against more *S. aureus* strains having also higher values of predicted probabilities. Such a difference indicates that the pyrrolic nitrogen atom is more adequate than sulfur; notice that sulfur leads to a slightly unfavorable increase of the polarizability (*DGTI*015), augmentation of the hydrophobicity (*DGTI*13 and *DGTI*18), and inadequate increase in molecular accessibility (*DGTI*19). Therefore, from a fragment-based point of view, the imidazole ring appears to add more versatility than the thiazole ring to the multi-strain antibacterial activity of the designed molecules.

When comparing MS-ASP-03 and MS-ASP-04, there are also differences. In this sense, MS-ASP-04 is predicted as active against more *S. aureus* strains than MS-ASP-03. This means that the phenol fragment in MS-ASP-04 seems to be more favorable than the indole in MS-ASP-03 in terms of positively contributing to the multi-strain antibacterial activity. Here, major factors contributing to the decrease in activity of MS-ASP-03 when compared to MS-ASP-04 are the increment of the polarizability (*DGTI*015) and the relative decrease of linearity of the molecule related to the presence of more cyclic fragments (*DGTI*10).

All the ideas mentioned above have evidence that the combination of the PTML-MLP model and the FBTD approach enables the design of seemingly novel molecular entities that potentially display multi-strain antibacterial activity. Nevertheless, we intended to gain more insights regarding the structural novelty of the designed molecules. To do so, we performed a search in large online databases such as ChEMBL [74,75,76], ZINC [77], eMolecules [78], and SureChEMBL [79]. We wanted to know if any of the structures of the four designed molecules were available in the aforementioned datasets; we applied a similarity cutoff of 80% to check for structural similarity, i.e., to analyze if the chemical structures of our designed molecules resemble the ones reported in the aforementioned databases. We could confirm that there were no molecules in those databases which are structurally similar to our designed molecules. This demonstrates that the joint use of our PTML model and the FBTD approach enables the generation of molecules, which, in addition to virtually exhibiting multi-strain antibacterial activity against *S. aureus*, also constitute new chemotypes for future synthesis and biological evaluation in the context of early antibacterial discovery.

### 2.3. Druglikeness Properties of the Designed Molecules

In addition to the novelty and encouraging potential antibacterial activity of the designed molecules against multiple *S. aureus* strains, we wanted to estimate their drug-like profiles. Here, we calculated a series of global physicochemical properties (Table 5); the software AlvaDesc v1.0.22 was employed to carry out such calculations [80].

The purpose underpinning the calculation of these global physicochemical properties check whether the designed molecules complied with well-established druglikeness guidelines such as Lipinski’s rule of five [81], Ghose’s filter [82], and Veber’s rules [83]. To do so, we compared the calculated values of the physicochemical properties of the designed molecules with the corresponding cutoff values established by the aforementioned druglikeness guidelines. The analysis of the results in Table 5 indicates that the four molecules comply with all the components of the three druglikeness guidelines; the only exception is one violation in the case of molecule MS-ASP-04 for Lipinski’s rule of five due to exceeding the number of hydrogen bond acceptors (nHAcc > 10); MS-ASP-04 complies with the other components of that rule. Therefore, we can conclude that the four designed molecules exhibit drug-like properties that are desirable in a molecule with acceptable oral bioavailability.

## 3. Materials and Methods

### 3.1. Data and Computation of the Graph-Theoretical Indices

A single file containing both the chemical and in vitro biological data was downloaded from the public web repository known as ChEMBL [74,75]. It is important to highlight that the chemical data were expressed as the simplified molecular-input line-entry system (SMILES) codes. The in vitro biological information contained the minimum inhibitory concentration (MIC) values experimentally determined for each molecule of the dataset against at least 1 out of 13 *S. aureus* strains (*bs*) through the broth microdilution method for the interval 16–24 h. In our dataset, certain MIC values appeared reported in nanomolar (nM) while for others, the measurement unit was µg/mL; we converted those values from µg/mL to nM by first dividing by the molar mass (MW) and then multiplying by the factor 10^6^.

We would like to emphasize that we applied two selection criteria to include these specific 13 *S. aureus*. strains in our study. On one side, these 13 *S. aureus* strains (*bs*) represent such diversity that captures a great phenotypic variability and clinical relevance. This means that the 13 *S. aureus* strains were selected to encompass a broad spectrum of drug resistance profiles and phenotypic variations. In this sense, among the 13 *S. aureus* strains, our study included (but was not limited to) methicillin-resistant (MRSA) and methicillin-susceptible (MSSA) strains, as well as multidrug-resistant (MDR) variants. This diversity ensured that the developed PTML-MLP model could predict antibacterial activity across a wide array of clinically relevant *S. aureus* strains. On the other hand, we only chose these 13 *S. aureus* strains because they were the ones for which a sufficient number of chemicals (>100) were experimentally tested.

Then, we eliminated all the entries for which the antibacterial activity values, units of measurement, or SMILES codes were missing. If a defined chemical was associated with different entries containing the same biological information (tested more than one time against the same *bs*), we kept only the entry of that molecule containing the lowest MIC value. Our final dataset contained 11,643 cases. Each case was labeled as active [*ABi*(*bs*) = 1] or inactive [*ABi*(*bs*) = −1]. In this sense, if for a defined case (a molecule assayed against a specific *S. aureus* strain), MIC ≤ 7000 nM, that case was labeled as activity; otherwise, the case was labeled as inactive. Notice that *ABi*(*bs*) was a binary variable indicating the antibacterial activity of the *i*th case against a defined *S. aureus* strain (*bs*).

It is important to point out that such antibacterial activity cutoff (MIC ≤ 7000 nM) is more rigorous than the one reported in high throughput screening experiments for the identifications of antibacterial molecules (MIC ≤ 10,000 nM) [84]. Consequently, the use of such a rigorous cutoff can enable the creation of any future machine learning model with the ability to search for chemicals virtually exhibiting potent antibacterial activity. Furthermore, the aforementioned activity cutoff prevented (as much as it was possible) the excessive imbalance between the number of chemicals labeled as active and the number of those annotated as inactive.

The dataset was divided into two subsets named training and test. These subsets comprised 75% and 25% of the dataset, respectively [37,45]. In this sense, for a given *S. aureus* strain, the cases/molecules were sorted by considering their increasing MIC values. After performing the ordering, the first three molecules/cases were annotated to belong to the training set while the fourth was labeled to belong to the test set. This procedure was repeated to label all the compounds tested against the same *S. aureus* strain, being subsequently applied to each *S. aureus* strain in the dataset (Supplementary Information S1).

The SMILES codes of the 11,643 cases were stored in *.txt file. Then, the MODESLAB software (version 1.5) was employed to calculate some families of topological indices (*TIs*) [85,86,87]. These *TIs* were spectral moments of the bond (edge) adjacency matrix [*SM*(*A*)*m*], vertex (atom)-based and vertex valence connectivity indices [*X*(*GF*)*k* and *Xv*(*GF*)*k*, respectively], bond (edge) connectivity indices [*e*(*GF*)*k*], and the shape indices [*Kt and K*(*Alpha*)t, with *t* assuming values from 1 to 3]. For the case of the *TIs* symbolized as *SM*(*A*)*m*, the letter *m* is the order, i.e., the maximum number of bonds (without considering bond order) that a fragment can have when calculating the *SM*(*A*)*m*. At the same time, “(*A*)” is an atomic or bond physicochemical property such as bond standard distance (*Std*), bond dipole moment (*Dip*), atomic hydrophobicity (*Hyd*), atomic polar surface area (*Psa*), atomic molar refractivity (*Mol*), van der Waals radius (*Van*), atomic Gasteiger–Marsili charges (*Gas*), an atomic weight (*Ato*), as well as Abraham solvation properties such as excess of molar refractivity (*AR2*), dipolarity/polarizability (*Api2H*), hydrogen bond basicity (*AB20*), and the atomic contribution to the hexadecane/gas phase partition coefficient (*logL16*) [88,89,90,91]. For the case of the *TIs* symbolized as *X*(*GF*)*k*, *Xv*(*GF*)*k*, and *e*(*GF*)*k*, “(*GF*)” refers to generic fragments (also known as subgraphs) and there are four types of them: paths (*P*), clusters (*C*), path-clusters (*PC*), and cycles/rings (*Ch*). The letter *k* is the order (the exact number of bonds without considering bond order) contained in each *GF* type. In addition to all the *TIs* mentioned here, we calculated normalization-like *TIs* (denoted as *NTIs*); for each molecule, each *NTI* was calculated as the quotient of a *TI* divided by *NB* (number of bonds without considering the bond order). The letter *t* for the case of *Kt and K*(*Alpha*)*t* has the same meaning as the letter *k*.

It is important to point out that 0D-descriptors (e.g., count descriptors), 1D-descriptors (such as molecular fingerprints), or 3D-descriptors (for instance, those derived from quantum chemical calculations) could have been used instead of the topological indices used in this work. However, other than the topological indices, any of the other chemical families suffer from at least one of the following disadvantages [86,87]. First, some of them do not have enough discriminatory power (0D-descriptors), particularly when used in large and heterogenous datasets such as the one used in the present work. Second, they may tend to either underestimate or overestimate the presence or absence of fragments or functional groups (e.g., molecular fingerprints). Third, the high computational cost associated with the calculations can be an issue and the assumption of conformation of minimum energy to characterize the 3D structure of the molecules can be misleading when creating predictive machine learning models because such conformations are rarely the active conformation; these are the cases of the 3D descriptors. In contrast, the topological indices mentioned above have been selected due to three key aspects. On one hand, they are very fast to calculate [86,87]. On the other hand, they can simultaneously consider the global physicochemical properties of the structures of the molecules as well as local features; the latter characteristic enables the topological indices to be expressed as a linear combination of the number of times in which the different subgraphs/fragments (eight) appear in the molecules [55,56,57,58,59,60,64,67,92]. Lastly, despite their 2D nature, topological indices can account for 3D aspects such as volume, molecular accessibility, and dihedral angles [61,66,93].

To characterize both the physicochemical and structural information of the molecules and the biological information (*S. aureus* strains against which each molecule was experimentally tested), we applied the Box–Jenkins approach using the following steps [37,45]:

(2) $$avg[GTI]bs=\frac{1}{n\left(bs\right)}\times \sum_{a=1}^{n\left(bs\right)}{GTI}_{a}\;$$

In Equation (2), *GTI* refers to the different graph-theoretical indices (both *TIs* or *NTIs*), *avg*[*GTI*]*bs* is an average-based term, and *n*(*bs*) is the number of molecules in the training set that were labeled as active while being experimentally assayed against the same *S. aureus* strain (*bs*). Then, a second mathematical formalism was applied to each molecule present in the dataset:

(3) $$D\left[GTI\right]bs=\left[\frac{GTI-avg\left[GTI\right]bs}{sd[GTI]}\right]\times \sqrt{p\left(bs\right)}\;$$

In Equation (3), *sd*[*GTI*] indicates the standard deviation calculated from the *GTI* values of the molecules annotated to belong to the training set. Also, *p*(*bs*) is an a priori probability of finding a molecule considered active against a defined *S. aureus* strain. The *D*[*GTI*]*bs* descriptors are known as the multi-label graph-theoretical indices and they simultaneously consider both the chemical structure of the molecules and the multiple labels of the element *bs* (different *S. aureus* strains). We would like to emphasize that multi-label graph-theoretical indices. Consequently, as inputs of a PTML model, the *D*[*GTI*]*bs* descriptors will enable any molecule for its antibacterial activity to be predicted 13 times (1 per each of the 13 *S. aureus* strains reported in the present study).

### 3.2. Creation, Performance Analysis, and Applicability Domain of the PTML-MLP Model

To select the most appropriate *D*[*GTI*]*bs* descriptors, the computer program named IMMAN (version 1.0) was used [94]. This software was employed to calculate three information indices, namely differential Shannon entropy [95], gain ratio [96], and symmetric uncertainty [97]. In doing so, the geometric mean values (*GMVs*) of these information indices were calculated for each *D*[*GTI*]*bs* descriptor; the highest discriminant power corresponded to those *D*[*GTI*]*bs* descriptors with the highest *GMVs*. After ranking the *D*[*GTI*]*bs* descriptors according to their decreasing *GMVs*, we used the software STATISTICA (v13.5.0.17) to perform a redundancy analysis [98]. This software enabled the calculation of the pair-wise Pearson’s correlation coefficient (*PCC*); we only kept those *D*[*GTI*]*bs* descriptors with *PCC* values in the range −0.7 < *PCC* < 0.7.

We also used STATISTICA to search for the best PTML-MLP model where the *D*[*GTI*]*bs* descriptors were employed as inputs. In doing so, we first compared the size of the dataset used in previous PTML models with the size of the present dataset; this allowed us to estimate the potential number of non-redundant *D*[*GTI*]*bs* descriptors (complying with the condition −0.7 < *PCC* < 0.7) to be used as inputs when searching for the best PTML-MLP model. We concluded that 20 to 25 *D*[*GTI*]*bs* descriptors would be enough to obtain a PTML-MLP model that had both acceptable statistical quality and good interpretability [37,45]. To search for the best PTML-MLP model (best MLP network), we used a default configuration for the setting parameters (e.g., number of networks to be trained, type of activation functions in the hidden and output layers, etc.) [37,45]; the only exceptions were the minimum and maximum numbers of neurons in the hidden layer, which, by considering Equation (1), were set to be 40 and 100, respectively. When analyzing different MLP networks to select the best one (PTML-MLP model), we checked different global statistical indices such as sensitivity (*Sn*), specificity (*Sp*), and the normalized Matthew’s correlation coefficient (*MCC*) [99]. In any case, the selection of the best MLP network was based on the analysis of the local sensitivities and specificities [*Sn*(*bs*) and *Sp*(*bs*), respectively] in both training and test sets [37,45], which depended on each *S. aureus* strain (*bs*). Therefore, the PTML-MLP model was the MLP network exhibiting the highest values of *Sn*(*bs*) and *Sp*(*bs*). Concerning the applicability domain (AD) of the PTML-MLP model, we employed a modification of the descriptor’s space (also known as the bounding box) approach [37,45].

## 4. Conclusions

Novel and more efficient antibacterial agents are urgently needed to fight against infections and the emergence of MDR caused by *S. aureus*. Consequently, at the early drug discovery level, computational approaches should focus on accelerating the discovery and/or design of chemicals with the potentiality to simultaneously target different *S. aureus* strains of varying degrees of drug resistance. The findings of the present work demonstrate that it is possible to interpret a PTML model through the use of the FBTD approach to gain insights into the physicochemical properties and structural requirements that can be responsible for the multi-strain antibacterial activity; this enabled the design of new chemicals displaying great versatility and potency against multiple *S. aureus* strains. The designed molecules theoretically exhibit great versatility as multi-strain inhibitors of *S. aureus* strains with different degrees of resistance to current antibiotics, and therefore, they could constitute new chemical entities to undergo future synthesis and biological evaluation with the potential to tackle MDR associated with *S. aureus*. The unified computational methodology combining PTML modeling and FBTD envisages encouraging opportunities for the generation of new chemotypes with multi-strain antibacterial activity, which could be extended to other bacterial pathogens and even beyond antibiotics research.

## Figures and Tables

**Table 1:** Symbols and definitions of the *D*[*GTI*]*bs* descriptors present in the PTML-MLP model.

Code ^a,b^	Symbology	Definition
*DGTI*01	*D*[*SM*(*Psa*)1]*bs*	Multi-label graph-theoretical index based on the bond spectral moment of order 1 weighted by the atomic polar surface area.
*DGTI*02	*D*[*SM*(*Psa*)5]*bs*	Multi-label graph-theoretical index based on the bond spectral moment of order 5 weighted by the atomic polar surface area.
*DGTI*03	*D*[*SM*(*Mol*)1]*bs*	Multi-label graph-theoretical index based on the bond spectral moment of order 1 weighted by the atomic molar refractivity.
*DGTI*04	*D*[*X*(*C*)3]*bs*	Multi-label graph-theoretical index based on atom connectivity of order 3 involving only cluster subgraphs.
*DGTI*05	*D*[*e*(*Ch*)3]*bs*	Multi-label graph-theoretical index based on bond connectivity of order 3 involving only cycle (ring) subgraphs.
*DGTI*06	*D*[*e*(*Ch*)4]*bs*	Multi-label graph-theoretical index based on bond connectivity of order 4 involving only cycle (ring) subgraphs.
*DGTI*07	*D*[*e*(*Ch*)5]*bs*	Multi-label graph-theoretical index based on bond connectivity of order 5 involving only cycle (ring) subgraphs.
*DGTI*08	*D*[*e*(*C*)6]*bs*	Multi-label graph-theoretical index based on bond connectivity of order 6 involving only cluster subgraphs.
*DGTI*09	*D*[*e*(*Ch*)6]*bs*	Multi-label graph-theoretical index based on bond connectivity of order 6 involving only cycle (ring) subgraphs.
*DGTI*10	*D*[*K*(*Alpha*)3]*bs*	Multi-label graph-theoretical index of order 3 based on molecular shape involving only path subgraphs.
*DGTI*11	*D*[*NSM*(*Std*)2]*bs*	Multi-label normalized graph-theoretical index based on the bond spectral moment of order 2 weighted by the standard bond distance.
*DGTI*12	*D*[*NSM*(*Dip*)7]*bs*	Multi-label normalized graph-theoretical index based on the bond spectral moment of order 7 weighted by the bond dipole moment.
*DGTI*13	*D*[*NSM*(*Hyd*)1]*bs*	Multi-label normalized graph-theoretical index based on the bond spectral moment of order 1 weighted by the atomic hydrophobicity.
*DGTI*14	*D*[*NSM*(*Hyd*)6]*bs*	Multi-label normalized graph-theoretical index based on the bond spectral moment of order 6 weighted by the atomic hydrophobicity.
*DGTI*15	*D*[*NSM*(*Mol*)2]*bs*	Multi-label normalized graph-theoretical index based on the bond spectral moment of order 2 weighted by the atomic molar refractivity.
*DGTI*16	*D*[*NSM*(*Van*)2]*bs*	Multi-label normalized graph-theoretical index based on the bond spectral moment of order 2 weighted by the van der Waals radius.
*DGTI*17	*D*[*NSM*(*Ato*)4]*bs*	Multi-label normalized graph-theoretical index based on the bond spectral moment of order 4 weighted by the atomic weight.
*DGTI*18	*D*[*NSM*(*logL16*)1]*bs*	Multi-label normalized graph-theoretical index based on the bond spectral moment of order 1 weighted by the atomic contribution to the hexadecane/gas phase partition coefficient.
*DGTI*19	*D*[*NXv*(*P*)2]*bs*	Multi-label normalized graph-theoretical index based on valence atom connectivity of order 2 involving only path subgraphs.
*DGTI*20	*D*[*NXv*(*Ch*)6]*bs*	Multi-label normalized graph-theoretical index based on valence atom connectivity of order 6 involving only cycle (ring) subgraphs.
*DGTI*21	*D*[*Ne*(*P*)1]*bs*	Multi-label normalized graph-theoretical index based on bond connectivity of order 1 involving only path subgraphs.

^a^ These are the codes for the different *D*[*GTI*]*bs* descriptors, which, for the sake of simplicity, will be used instead of the symbols when describing the physicochemical and structural interpretations. ^b^ For the *D*[*GTI*]*bs* descriptors containing the notation “*SM*”, the order (described in the Material and Methods section as *m*) represents the maximum number of bonds (without counting bond order) that a fragment can have. Also, for the *D*[*GTI*]*bs* descriptors containing the notations “*X*”, “*Xv*”, and “*e*”, the order (also mentioned in Section 3 as *k*) represents the exact number of bonds (without counting bond order) present in a fragment. For the case of the *D*[*GTI*]*bs* descriptors containing the notation “*K*(”, the order (also mentioned in Section 3 as *t*) has the same meaning as the letter *k*.

**Table 2:** Statistical quality of the PTML-MLP model in terms of global performance metrics.

SYMBOLS ^a^	Training Set	Test Set
*N*_Active_	3722	1232
*CC*_Active_	3137	980
*Sn*	84.28%	79.55%
*N*_Inactive_	5016	1673
*CC*_Inactive_	4554	1417
*Sp*	90.79%	84.70%
*nMCC*	0.877	0.821

^a ^*N*_Active_—number of molecules/cases labeled as active; *N*_Inactive_—number of molecules/cases labeled as inactive; *CC*_Active_—number of molecules/cases correctly classified as active; *CC*_Inactive_—number of molecules/cases correctly classified as inactive; *Sn*—sensitivity (percentage of molecules/cases correctly classified as active); *Sp*—specificity (percentage of molecules/cases correctly classified as inactive); *nMCC*—normalized Matthews’ correlation coefficient.

**Table 3:** Tendency of variations of the *D*[*GTI*]*bs* descriptors estimated by calculating class-based arithmetic means.

Code ^a^	ARITHMETIC MEANS ^b^		Propensity ^c^
	Active	Inactive	
*DGTI*01	3.0465 × 10^−2^	−2.6581 × 10^−1^	Increase
*DGTI*02	1.0490 × 10^−2^	−1.0355 × 10^−1^	Increase
*DGTI*03	3.9720 × 10^−2^	−3.6811 × 10^−1^	Increase
*DGTI*04	4.7883 × 10^−2^	−3.5375 × 10^−1^	Increase
*DGTI*05	3.5900 × 10^−2^	−2.1759 × 10^−1^	Increase
*DGTI*06	1.5332 × 10^−2^	−3.4821 × 10^−2^	Increase
*DGTI*07	1.6622 × 10^−2^	−6.0985 × 10^−2^	Increase
*DGTI*08	3.0394 × 10^−2^	−1.4827 × 10^−1^	Increase
*DGTI*09	1.1054 × 10^−7^	−8.2182 × 10^−2^	Increase
*DGTI*10	2.0196 × 10^−2^	−1.6413 × 10^−1^	Increase
*DGTI*11	4.7825 × 10^−2^	−2.8640 × 10^−1^	Increase
*DGTI*12	2.4487 × 10^−2^	−4.5096 × 10^−2^	Increase
*DGTI*13	−2.3486 × 10^−2^	1.4339 × 10^−1^	Decrease
*DGTI*14	2.4933 × 10^−2^	−1.4037 × 10^−1^	Increase
*DGTI*15	−9.2336 × 10^−3^	1.2918 × 10^−1^	Decrease
*DGTI*16	1.8411 × 10^−2^	−8.7712 × 10^−2^	Increase
*DGTI*17	7.1753 × 10^−3^	6.9784 × 10^−2^	Decrease
*DGTI*18	−1.6442 × 10^−2^	2.2357 × 10^−1^	Decrease
*DGTI*19	2.0916 × 10^−2^	−9.4055 × 10^−3^	Increase
*DGTI*20	−3.1756 × 10^−3^	5.0729 × 10^−2^	Decrease
*DGTI*21	−3.0244 × 10^−2^	2.5218 × 10^−1^	Decrease

^a^ The codes are the same as those represented in Table 1. ^b^ The class-based arithmetic means were calculated according to an approach reported by Speck-Planche and co-workers in recent reports [37,45,52]. ^c^ It reflects how the value of a *D*[*GTI*]*bs* descriptor should vary (increase or decrease) to enhance the multi-strain antibacterial activity against different *S. aureus* strains.

**Table 4:** Multi-strain antibacterial activity of the designed molecules predicted by the PTML-MLP model.

PREDICTION RESULTS ^a^					
ID	*S. aureus* Strains (*bs*)	ProbAct	ID	*S. aureus* Strains (*bs*)	ProbAct
MS-ASP-01	*S. aureus * (ATCC 13709)	85.65%	MS-ASP-03	*S. aureus * (ATCC 13709)	77.52%
MS-ASP-01	*S. aureus* (ATCC 25923)	63.27%	MS-ASP-03	*S. aureus* (ATCC 25923)	46.48%
MS-ASP-01	*S. aureus* (ATCC 29213)	86.16%	MS-ASP-03	*S. aureus* (ATCC 29213)	77.05%
MS-ASP-01	*S. aureus* (ATCC 33591)	87.34%	MS-ASP-03	*S. aureus* (ATCC 33591)	76.69%
MS-ASP-01	*S. aureus* (ATCC 33592)	83.73%	MS-ASP-03	*S. aureus* (ATCC 33592)	79.72%
MS-ASP-01	*S. aureus* (ATCC 43300)	86.41%	MS-ASP-03	*S. aureus* (ATCC 43300)	76.44%
MS-ASP-01	*S. aureus* (ATCC 700699)	86.27%	MS-ASP-03	*S. aureus* (ATCC 700699)	78.84%
MS-ASP-01	*S. aureus* (MDR)	84.79%	MS-ASP-03	*S. aureus* (MDR)	75.52%
MS-ASP-01	*S. aureus* (Methicillin-Resistant)	87.47%	MS-ASP-03	*S. aureus* (Methicillin-Resistant)	83.15%
MS-ASP-01	*S. aureus* (Methicillin-Susceptible)	87.90%	MS-ASP-03	*S. aureus* (Methicillin-Susceptible)	85.56%
MS-ASP-01	*S. aureus* (N315)	44.73%	MS-ASP-03	*S. aureus* (N315)	43.31%
MS-ASP-01	*S. aureus* (RN4220)	83.14%	MS-ASP-03	*S. aureus* (RN4220)	79.88%
MS-ASP-01	*S. aureus* (USA300)	78.13%	MS-ASP-03	*S. aureus* (USA300)	62.99%
MS-ASP-02	*S. aureus * (ATCC 13709)	72.88%	MS-ASP-04	*S. aureus * (ATCC 13709)	78.38%
MS-ASP-02	*S. aureus* (ATCC 25923)	38.92%	MS-ASP-04	*S. aureus* (ATCC 25923)	60.93%
MS-ASP-02	*S. aureus* (ATCC 29213)	52.96%	MS-ASP-04	*S. aureus* (ATCC 29213)	83.35%
MS-ASP-02	*S. aureus* (ATCC 33591)	62.78%	MS-ASP-04	*S. aureus* (ATCC 33591)	82.33%
MS-ASP-02	*S. aureus* (ATCC 33592)	79.41%	MS-ASP-04	*S. aureus* (ATCC 33592)	84.08%
MS-ASP-02	*S. aureus* (ATCC 43300)	51.29%	MS-ASP-04	*S. aureus* (ATCC 43300)	81.72%
MS-ASP-02	*S. aureus* (ATCC 700699)	66.96%	MS-ASP-04	*S. aureus* (ATCC 700699)	86.26%
MS-ASP-02	*S. aureus* (MDR)	75.57%	MS-ASP-04	*S. aureus* (MDR)	85.97%
MS-ASP-02	*S. aureus* (Methicillin-Resistant)	61.41%	MS-ASP-04	*S. aureus* (Methicillin-Resistant)	86.32%
MS-ASP-02	*S. aureus* (Methicillin-Susceptible)	66.06%	MS-ASP-04	*S. aureus* (Methicillin-Susceptible)	86.69%
MS-ASP-02	*S. aureus* (N315)	38.15%	MS-ASP-04	*S. aureus* (N315)	42.85%
MS-ASP-02	*S. aureus* (RN4220)	66.40%	MS-ASP-04	*S. aureus* (RN4220)	75.70%
MS-ASP-02	*S. aureus* (USA300)	44.35%	MS-ASP-04	*S. aureus* (USA300)	75.94%

^a^ ProbAct refers to the probability (expressed in percentage) predicted by the PTML-MLP model for a molecule to be classified as active against a specific *S. aureus* strain.

**Table 5:** Global physicochemical properties calculated for the designed molecules.

ID ^a^	MW	nHDon	nHAcc	MlogP	AlogP	nAT	AMR	NRB	PSA
MS-ASP-01	448.82	2	10	2.3186	3.4572	43	103.52	4	109.70
MS-ASP-02	465.87	1	10	2.2549	4.0255	42	107.72	4	122.15
MS-ASP-03	466.47	1	10	2.6201	3.1361	51	113.7404	5	105.48
MS-ASP-04	443.43	1	11	2.5295	2.5752	48	104.348	5	109.92

^a^ The symbols of the global physicochemical properties are MW—molecular weight (expressed in daltons); nHDon—number of atoms capable of acting as hydrogen bond donors; nHAcc—number of atoms capable of acting as hydrogen bond acceptors; MlogP—logarithm of the partition coefficient (octanol/water) calculated by using the Moriguchi’s approach; AlogP—logarithm of the partition coefficient (octanol/water) calculated by using the Ghose–Crippen’s approach; nAT—number of atoms; AMR—molar refractivity (expressed in cm^3^/mol) calculated by using the Ghose-Crippen’s approach; NRB—number of rotatable bonds; PSA—topological polar surface area (expressed in Å^2^) calculated from functional groups containing nitrogen, oxygen, sulfur, and phosphorus.

## Data Availability

Data are provided within the manuscript and Supplementary Information Files.

## Supplementary Materials

The following supporting information can be downloaded at: https://www.mdpi.com/article/10.3390/ph18020196/s1, Supplementary Information S1: Topological indices (*TIs* and *NTIs*), averages, and standard deviation values *sd*[*GTI*]. Supplementary Information S2: *D*[*GTI*]*bs* descriptors, classification results, local metrics, and applicability domain. Supplementary Information S3: Topological indices (*TIs* and *NTIs*), *D*[*GTI*]*bs* descriptors, classification results, and applicability domain for the designed molecules.
